# Neural Correlates of Response Inhibition and Conflict Control on Facial Expressions

**DOI:** 10.3389/fnhum.2017.00657

**Published:** 2018-01-09

**Authors:** Tongran Liu, Tong Xiao, Jiannong Shi

**Affiliations:** ^1^CAS Key Laboratory of Behavioral Science, Institute of Psychology, Chinese Academy of Sciences, Beijing, China; ^2^Department of Psychology, University of Chinese Academy of Sciences, Beijing, China; ^3^Natural Language Processing Laboratory, Northeastern University, Liaoning, China; ^4^Department of Learning and Philosophy, Aalborg University, Aalborg, Denmark

**Keywords:** response inhibition, conflict control, emotion regulation, facial expressions, event-related potential

## Abstract

Response inhibition and conflict control on affective information can be regarded as two important emotion regulation and cognitive control processes. The emotional Go/Nogo flanker paradigm was adopted and participant’s event-related potentials (ERPs) were analyzed to investigate how response inhibition and conflict control interplayed. The behavioral findings revealed that participants showed higher accuracy to identify happy faces in congruent condition relative to that in incongruent condition. The electrophysiological results manifested that response inhibition and conflict control interplayed during the detection/conflict monitoring stage, and Nogo-N2 was more negative in the incongruent trials than the congruent trials. With regard to the inhibitory control/conflict resolution stage, Nogo responses induced greater frontal P3 and parietal P3 responses than Go responses did. The difference waveforms of N2 and parietal P3 showed that response inhibition and conflict control had distinct processes, and the multiple responses requiring both conflict control and response inhibition processes induced stronger monitoring and resolution processes than conflict control. The current study manifested that response inhibition and conflict control on emotional information required separable neural mechanisms during emotion regulation processes.

## Introduction

Emotion regulation is essential for individual’s social life (Gross, 2015), and it refers to the attempts to influence which emotions to have, when to have them, and how to experience or express them (Gross, 1998). According to the process model of emotion regulation (Gross, 1998), there are five essential emotion regulation strategies: situation selection, situation modification, attentional deployment, cognitive change and response modulation. Suppression (inhibition) has been attributed as one of the best forms of response modulation during emotional regulation, which refers to the efforts to inhibit individual’s emotion-expressive behavior (Gross, 2015). Response inhibition to affectively salient stimuli can provide more insights into the emotion-modulated inhibition control processes (Schulz et al., 2007; Chiu et al., 2008; Albert et al., 2010, 2012). It has also been found that emotional processing on the erotic and painful information can impair individual’s following inhibitory control processes (Yu et al., 2012, 2015), and the threatening information can improve inhibitory performances due to the enhancement of perceptual and cognitive processes (Senderecka, 2016). Moreover, emotional conflict control is regarded as playing an important role in monitoring affective conflict situations and modulating the influence of emotional and social distractors on behavior (Botvinick et al., 2001), and it relates to attentional deployment during emotion regulation by directing selective attention with the goal of affecting individual’s emotional response (Gross et al., 2011a,b; Etkin et al., 2015; Gross, 2015; Sheppes et al., 2015). The main aim of current study was to investigate how perceived emotional information modulated the interaction between response inhibition and conflict control processes.

Response inhibition is broadly investigated by the Go/Nogo paradigm with the requirement of executing responses to one type of stimuli (Go response) and withholding responses to the other type (Nogo response), and response inhibition on affective information might relate to suppression on improper emotions during emotion regulation (Ladouceur et al., 2006; Schacht et al., 2009; Frischen et al., 2012; Yu et al., 2014). In the Go/Nogo task, a negative event-related potentials (ERPs) component, the Nogo-N2 with fronto-central neural generation is elicited in 250–350 ms time window after the occurrence of Nogo stimuli; the N2 component reflects the detection of conflict between inhibition requirement and response execution (Nieuwenhuis et al., 2003; Donkers and van Boxtel, 2004; Lo et al., 2013; Ocklenburg et al., 2013). It has also been found that frontal beta power relates to the neurodevelopment of inhibitory control during early childhood (Lo et al., 2013). Some studies have shown that the amplitude of Nogo-N2 cannot be affected by the emotional valence of information (Chiu et al., 2008; Todd et al., 2008; Yu et al., 2009; Albert et al., 2010; Zhang and Lu, 2012), however, some other studies reported that arousing negative information or highly unpleasant information would induce more negative Nogo-N2 responses compared with neutral and pleasant information (Albert et al., 2012; Yuan et al., 2012).

Another essential ERP component during response inhibition is the Nogo-P3 occurring in 300–600 ms time window after the onset of the Nogo stimuli over fronto-central brain areas (Kiefer et al., 1998), and it is associated with successful motor suppression and the evaluation of the inhibition outcome (Bruin et al., 2001). Relative to the Nogo-P3, the Go-P3 is in response to Go stimuli and distributed mainly over parietal areas, and it relates to execution rather than inhibition (Tekok-Kilic et al., 2001). In the emotional Go/Nogo tasks, Nogo-P3 responses to emotional information were larger and faster than Go emotional responses (Zhang and Lu, 2012). Varied findings have been reported about the modulation of emotional valence on the Nogo-P3 responses. Albert et al. (2010, 2012) reported that Nogo-P3 is greater to pleasant information compared to unpleasant information, which was not observed in Yuan et al.’s (2012) study. Zhang and Lu (2012) observed that the Nogo-P3 responses to both negative and positive expressions were greater than that to neutral expressions, however, Chiu et al. (2008) found that the Nogo-P3 could not be modulated by the emotional valence of the stimuli.

Conflict control, which is widely measured by the flanker paradigm (Eriksen and Eriksen, 1974) relates to the processes of monitoring the conflicts in perceptual inputs or between required responses and individual’s preferred responses (Botvinick et al., 2001), and conflict control on affective information relates to the monitoring and resolution processes on affective conflicts (Albert et al., 2010). Several electrophysiological and brain imaging studies have manifested that conflict N2 is sensitive to conflict monitoring with the neural generators of frontal regions, such as anterior cingulated cortex (ACC) and prefrontal cortex (van Veen and Carter, 2002a,b; Ullsperger et al., 2005; Folstein and Van Petten, 2008), and N2 amplitudes are more negative for incongruent trials compared to congruent trials (van Veen and Carter, 2002a,b). The P3 responses are associated with conflict resolution and allocation of attentional control (Hillman et al., 2009a,b; Clayson and Larson, 2011), and the incongruent trials induce larger P3 than the congruent trials (Hillman et al., 2009a,b). Previous behavioral and electrophysiological studies showed that response speed was faster in emotional congruent trials than emotional incongruent trials, and happy faces with sad distracters induced more negative N2 amplitudes than happy faces flanked by identical faces during conflict monitoring stage (Fenske and Eastwood, 2003; Liu et al., 2013); while during the conflict resolution stage, happy faces in the incongruent trials elicited slower P3 responses compared to that in the congruent trials, and the attentional control on sad faces in the incongruent trials induced larger P3 responses than that in the congruent trials (Fenske and Eastwood, 2003; Liu et al., 2013). In the same line, Ochsner et al. (2009) adopted functional magnetic resonance imaging (fMRI) technology to investigated conflict control on affective words and reported that bilateral dorsal ACC were strongly activated in the affective incongruent condition.

As introduced above, conflict control and response inhibition are two vital emotion regulation strategy processes (Zhang and Lu, 2012), and recent studies have investigated how these two processes interplayed in the non-emotional context (Bunge et al., 2002; Huizinga et al., 2006; Brydges et al., 2012, 2013). By using a Go/Nogo flanker task, Brydges et al. (2012) reported that maximum N2 responses distributed on midline electrodes over central areas for conflict control and midline electrodes over frontal areas for response inhibition, and they also found that N2 latencies were longer for conflict control than for responses inhibition. Two developmental studies also demonstrated the distinction of conflict control and response inhibition with the Go/Nogo flanker tasks, and it has been reported that these two processes induced distinct brain areas in children and adults (Bunge et al., 2002; Brydges et al., 2013). Brydges et al. (2013) further illustrated that N2 responses gradually frontally distributed with age for response inhibition, and the N2 latencies and amplitudes became shortened or decreased with age development; however, with regard to conflict control, the significant N2 effect was only shown in adults but not in children.

Moreover, emotional stimuli, such as facial expressions, might carry essential social-emotional information, and the accurate and proper detection, perception, management and regulation on facial expressions were essential for individual’s emotion regulation (Calder and Young, 2005). However, it is less known about the interaction and/or distinction between response inhibition and conflict control in emotional contexts. Hence, the main aim of current study was to investigate the interaction between response inhibition and conflict control on facial expressions. It was hypothesized that the situation that required both Nogo responses and conflict control would require greater N2 and P3 activation compared to other conditions, and response inhibition and conflict control in emotional context induced different brain activities.

## Materials and Methods

### Ethics Statement

This study was approved by the Ethics Committee of Institute of Psychology, Chinese Academy of Sciences and was conducted according to the principles expressed in the Declaration of Helsinki. All the participants provided written informed consent prior to their participation.

### Participants

Twenty-eight participants (16 females and 12 males, 21–30 years, mean age: 25.4 years) were paid 100 RMB for participating in the current ERP experiment. All the participants reported normal or corrected-to-normal visual acuity and were naïve to the purpose of the experiment. None of them reported neurological or psychiatric problems.

### Materials and Procedure

A revised emotional Go/Nogo flanker task was adopted in the present study, which was similar to previous non-emotional Go/Nogo flanker tasks (Bunge et al., 2002; Brydges et al., 2012, 2013). Each stimulus consisted of five emotional faces with one central target face (either happy or fearful faces) and two faces (either happy or fearful faces) on the bilateral sides of target face. The expressional faces were from 10 Chinese models (five males and five females, ages from 25 years to 29 years), and all the images had similar luminance. Prior to formal experiment, the normative 9 point scale ratings were carried by another six volunteers to assess the valence and arousal of each facial expression image. For the valence rating, the *t*-test showed that happy images (*M* = 5.64, SD = 0.31) had higher scores than fearful images (*M* = 1.54, SD = 0.29; *p* < 0.005). For the arousal rating, there were no significant differences between happy (*M* = 6.22, SD = 0.51) and fearful images (*M* = 6.31, SD = 0.54) on the arousal scores (*p* > 0.05). After the formal study, all the 28 participants were required to evaluate the valence and arousal of facial expression images, and happy faces (*M* = 5.69, SD = 0.28) were rated higher than fearful faces on valence values (*M* = 1.57, SD = 0.26; *p* < 0.005), and happy faces (*M* = 6.24, SD = 0.34) and fearful faces (*M* = 6.29, SD = 0.33) obtained similar arousal value scores (*p* > 0.05).

The five faces in each stimulus were from one identical model, and each stimulus was displayed on a light gray screen of a 17-inch computer monitor (1024 × 768 at 100 Hz) with visual angle of 3.8° horizontally and 1° vertically. Based on the congruency between the target face and bilateral distractor faces, the congruent conditions were the stimuli of happy target face flanked by happy faces [HHHHH] and fearful target face flanked by fearful faces [FFFFF], and the incongruent conditions were the stimuli of happy target face with fearful distractors [FFHFF] and fearful target face with happy distractors [HHFHH].

**Figure 1:**
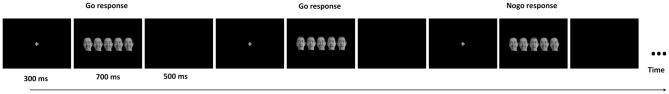
The diagram of experimental stimuli and procedure. In this block, participants were instructed to focus on the central target faces and ignore the bilateral flanked faces, and they were required to execute Go responses to happy faces and Nogo responses to fearful faces. According to the combination rules between Go/Nogo paradigm and flanker paradigm, there were four types of trials, Go_Congruent trials, Go_Incongruent trials, Nogo_Congruent trials and Nogo_Incongruent trials.

During the formal ERP experiment, participants were seated on a comfortable chair with a straight angle to the center of the computer monitor, and the viewing distance was 65 cm. At the beginning of each trial, there was a fixation “+” for 300 ms, and each stimulus was displayed for 700 ms, followed by a blank for 500 ms. The current task contained eight experimental blocks, and in all blocks participants were required to concentrate on the target face and ignore the bilateral faces. In four blocks, participants were required to press the response button to the central fearful face (Go response) and withhold their responses to the central happy face (Nogo response). In the other four blocks, participants were instructed to press the response button to the central happy face (Go response) and withhold their responses to the central fearful face (Nogo response). Therefore, according to the interaction between the congruency of stimuli (congruent, incongruent) and the Go/Nogo responses (Go, Nogo), there were four types of conditions: Go_congruent condition, Go_incongruent condition, Nogo_congruent condition and Nogo_incongruent condition, and the ratios of numbers for each type of conditions were 2:2:1:1. Each block consisted of 40 Go_congruent trials, 40 Go_incongruent trials, 20 Nogo_congruent trials and 20 Nogo_incongruent trials. After each block, participants were allowed to have 2–3 min break. For each condition (Go_Congurent, Go_Incongruent, Nogo_Congruent and Nogo_Incongruent), half of stimuli were male faces, and the other half were female faces, which ensured that the perception of gender information was identical in each condition. Figure 1 shows the sample of experimental stimuli and procedure.

### ERP Recording and Analysis

Electroencephalograms (EEG) were recorded from 64 scalp electrodes that were placed according to 10–20 system locations, and four bipolar electrodes monitoring horizontal and vertical EOG (HEOG and VEOG) were positioned on the outer canthi of two eyes and in the inferior and superior areas of left eye, respectively. The skin resistance of each electrode was adjusted under 5 kΩ. EEG was continuously recorded at a sample rate of 1000 Hz with online band-pass filter at 0.05–100 Hz using nose reference. EEG signal was epoched with 100 ms prior to and 900 ms after the stimulus onset, and the pre-stimulus 100 ms interval was used for baseline correction. Epochs contaminated by eye blinks, eye movements, or muscle potentials exceeding ±35 μV at any electrode were excluded. ERPs were further Zero Phase Shift filtering offline (bandwidth, 1–30 Hz, slope, 24 dB/octave). The numbers of artifact-free trials for all the average conditions were as follows. With average of 107 trials for happy Go_congruent condition (range from 78 to 154 trials), 103 trials for happy Go_incongruent condition (range from 79 to 140 trials), 55 trials for happy Nogo_congruent condition (range from 43 to 73 trials), 54 trials for happy Nogo_incongruent condition (range from 41 to 71 trials), 110 trials for fearful Go_congruent condition (range from 81 to 147 trials), 110 trials for fearful Go_incongruent condition (range from 80 to 150 trials), 52 trials for fearful Nogo_congruent condition (range from 40 to 66 trials), and 51 trials for fearful Nogo_incongruent condition (range from 42 to 64 trials). The numbers of trials that were included in analyses had similar proportions with raw structure of each type of stimuli, and these artifact-free ERP trial numbers were not affected by task types and/or facial expression conditions (*p*s > 0.05).

### Statistical Analyses on the Behavioral and ERP Data

#### Behavioral Data

The correct response rate (CRR) for Go responses was measured with numbers of correct responses to the target stimuli divided by total numbers of Go responses, and the commission error rate (CER) for Nogo responses was calculated with the numbers of commission responses to the non-target stimuli divided by numbers of the non-target stimuli. The parametric tests (Kolmogorov-Smirnov) have been conducted on the indexes of CRR and CER in each condition, and it is shown that all the CRRs and CERs are normally distributed. The CRR-Go and CER-Nogo were analyzed by 2 × 2 × 2 ANOVAs with the independent variables of Go/Nogo responses (Go, Nogo), Target expression (happy face, fearful face), and Congruency (congruent trial, incongruent trial). The mean reaction time (RT) of correct Go responses was analyzed by a 2 × 2 ANOVA with the independent variables of Target expression (happy face, fearful face) and Congruency (congruent trial, incongruent trial). The Greenhouse-Geisser correction was applied to estimate epsilon and correct the degree of freedom of the F-distribution if sphericity had been violated. *Post hoc* comparisons were calculated and adjusted by the Sidak test.

#### ERP Data

The peak amplitudes and latencies of N2 and P3 were analyzed by 2 × 2 × 2 ANOVAs with the independent variables of Target expression (happy face, fearful face), Congruency (congruent trial, incongruent trial), and Go/Nogo responses (Go response, Nogo response). The N2 was analyzed over the frontal and central areas (average of electrode sites of F3, Fz, F4, FC3, FCz, and FC4) during 210–350 ms intervals. The frontal P3 was analyzed with 450–650 ms time window over the frontal and central areas (average of electrode sites of F3, Fz, F4, FC3, FCz, and FC4), and the parietal P3 was analyzed in the time window of 400–650 ms over the central and parietal areas (average of electrode sites of C3, Cz, C4, CP3, CPz, CP4, P3, Pz and P4).

Furthermore, we also calculated the differences among the four types of conditions to get purified response inhibition responses, conflict control responses, and the multiple responses requiring both conflict control and response inhibition processes in the same task. Response inhibition responses = Congruent_Nogo trials − Congruent_Go trials; Conflict control responses = Incongruent_Go trials − Congruent_Go trials; and Multiple responses = Incongruent_Nogo trials − Congruent_Go trials. The difference amplitudes and latencies of N2 and P3 were analyzed by 2 × 3 ANOVAs with independent variables of Target expression (happy face, fearful face) and Response condition (Response inhibition, Conflict control and Multiple processes of response inhibition and conflict control). The Greenhouse-Geisser correction was applied to estimate epsilon and correct the degree of freedom of the F-distribution if sphericity had been violated. *Post hoc* comparisons were calculated and adjusted by the Sidak test.

## Results

### Behavioral Results

Means and standard deviations (SD) of CRRs for Go responses, CERs for Nogo responses, and RT of correct Go responses are presented in Table 1. For the RT, the main effect of Expression was significant (*F*_(1,27)_ = 8.2, *p* = 0.008 < 0.01, *η*^2^ = 0.24, Power = 0.75), and the identification of happy central face was faster relative to fearful face. For the CRR-Go responses, the main effect of Expression was significant, *F*_(1,27)_ = 9.57, *p* < 0.005, *η*^2^ = 0.26, Power = 0.85, and participants had higher accuracy when identifying happy faces than fearful faces. The interaction of Target expression × Congruency was significant (*F*_(1,27)_ = 3.92, *p* = 0.059, *η*^2^ = 0.13, Power = 0.48), and the identification of happy faces was more accurate within the congruent trials relative to incongruent trials (*p* < 0.003).

**Table 1:** Means and standard deviations (SD) of the correct response rates of Go responses (CRR-Go), the commission error rates of Nogo responses (CER-Nogo), and reaction time (RT) of correct Go responses (ms) in all conditions.

Congruency	Target expression	Go response		Nogo response
		CRR-Go	RT	CER-Nogo
Congruent	Fearful	0.87 ± 0.08	504 ± 33	0.12 ± 0.07
	Happy	0.93 ± 0.05	473 ± 41	0.11 ± 0.07
Incongruent	Fearful	0.87 ± 0.09	506 ± 32	0.13 ± 0.06
	Happy	0.92 ± 0.07	476 ± 41	0.14 ± 0.06

### ERP Results

**Figure 2:**
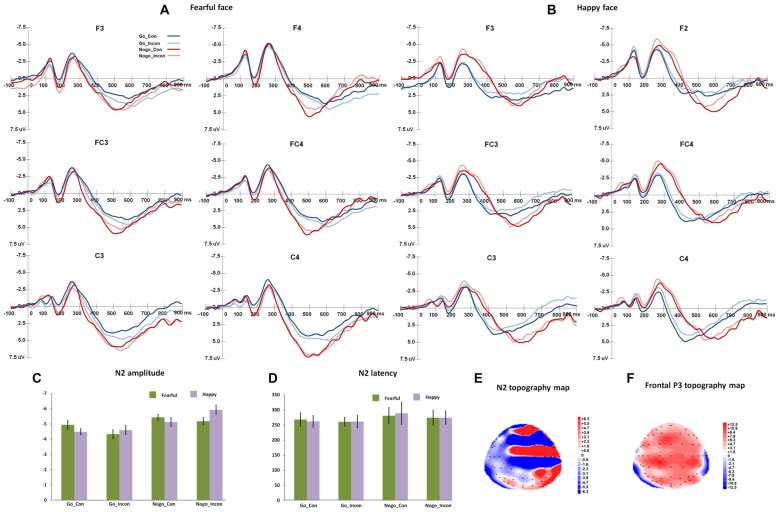
The N2 and frontal P3 responses in different conflict control and response inhibition conditions. **(A,B)** show the grand-average N2 responses to fearful target face and happy target face, respectively. **(C,D)** present the peak amplitudes and latencies and of N2 responses to fearful and happy target faces. **(E,F)** show the topography maps of N2 and frontal P3 responses, respectively.

**Figure 3:**
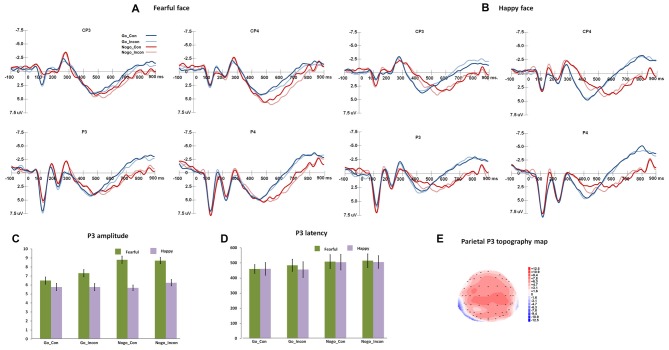
The P3 responses in different conflict control and response inhibition conditions. **(A,B)** show the grand-average P3 responses to fearful and happy faces over central-parietal and parietal areas, and the peak amplitudes and latencies of P3 are presented in **(C,D)**. **(E)** shows the topography map of parietal P3 responses.

Figure 2 shows the N2 and frontal P3 responses in all the conditions, and Figure 3 displays the parietal P3 responses in all the conditions.

### N2

For N2 peak latencies, the main effect of Go/Nogo was significant (*F*_(1,27)_ = 20.19, *p* < 0.001, *η*^2^ = 0.43, Power = 0.99), and Nogo responses induced slower N2 responses than Go responses. The main effect of Congruency was significant (*F*_(1,27)_ = 6.10, *p* < 0.05, *η*^2^ = 0.2), and N2 responses were faster in incongruent trials than in congruent trials.

For N2 peak amplitudes, the main effect of Go/Nogo was significant (*F*_(1,27)_ = 35.12, *p* < 0.001, *η*^2^ = 0.57, Power = 1.00), and Nogo responses elicited more negative N2 amplitudes than Go responses. The interaction between Congruency and Go/Nogo was significant (*F*_(1,27)_ = 7.97, *p* < 0.005, *η*^2^ = 0.23, Power = 0.78), and for Go responses, N2 amplitudes were more negative in congruent trials than that in incongruent trials (*p* < 0.05); for Nogo responses, N2 amplitudes were more negative in incongruent trials than in congruent trials (*p* < 0.05). The interaction of Expression × Congruency was significant (*F*_(1,27)_ = 8.12, *p* < 0.005, *η*^2^ = 0.23, Power = 0.78), and happy faces induced more negative N2 responses than fearful faces in incongruent trials (*p* < 0.05). The *post hoc* analyses also showed that fearful faces elicited more negative N2 amplitudes in the congruent trials than in the incongruent trials (*p* < 0.005), and happy faces induced more negative N2 amplitudes in the incongruent trials relative to congruent trials (*p* < 0.01).

For N2 difference latencies, the main effect of Response condition was significant (*F*_(2,54)_ = 8.50, *p* < 0.001, *η*^2^ = 0.24, Power = 0.91), and the *post hoc* analyses showed that multiple processes and response inhibition showed larger difference N2 latencies than conflict control (*p*s < 0.005). For N2 difference amplitudes, the main effect of Response condition was significant (*F*_(2,54)_ = 18.23, *p* < 0.005, *η*^2^ = 0.40, Power = 1.00), and the multiple processes and response inhibition had more negative difference N2 amplitudes than conflict control (*p*s < 0.005).

### Frontal P3

For frontal P3 latencies, the interaction between Expression and Go/Nogo was significant (*F*_(1,27)_ = 16.3, *p* < 0.001, *η*^2^ = 0.38, Power = 0.97), and for happy faces, frontal P3 was faster for Go response than Nogo response (*p* < 0.02); while for fearful faces, frontal P3 was faster for Nogo response than Go response (*p* < 0.001). For Go responses, happy targets induced faster frontal P3 than fearful targets (*p* < 0.003), and for Nogo responses, fearful targets induced faster frontal P3 than happy targets (*p* < 0.005). For the amplitudes of frontal P3, the main effect of Expression was significant, *F*_(1,27)_ = 12.32, *p* < 0.005, *η*^2^ = 0.32, Power = 0.92, and fearful targets induced larger frontal P3 responses than happy faces did. The main effect of Go/Nogo responses was also significant (*F*_(1,27)_ = 34.42, *p* < 0.001, *η*^2^ = 0.56, Power = 1.00), and the Nogo responses had greater frontal P3 than the Go responses did.

For the difference amplitude of frontal P3 responses, the main effect of Response condition was significant (*F*_(2,54)_ = 15.96, *p* < 0.001, *η*^2^ = 0.37, Power = 1.00), and the mixes responses and response inhibition had greater frontal P3 amplitude difference than conflict control condition (*p*s < 0.001). For the difference latencies of frontal P3, the main effect of Expression was significant (*F*_(1,27)_ = 18.23, *p* < 0.001, *η*^2^ = 0.40, Power = 1.00), and happy faces had positive frontal P3 difference and fearful faces had negative frontal P3 difference. The interaction between Expression and Response condition was significant (*F*_(2,54)_ = 16.56, *p* < 0.001, *η*^2^ = 0.38, Power = 0.99), and in the response inhibition condition and the multiple condition, the frontal-P3 difference response was positive for happy faces, while it was negative for fearful faces. For fearful faces, response inhibition and multiple conditions had negative frontal P3 difference, while conflict control had positive frontal P3 difference.

### Parietal P3

For parietal P3 peak latencies, the main effect of Go/Nogo was significant, *F*_(1,27)_ = 124.06, *p* < 0.001, *η*^2^ = 0.82, Power = 1.00, and Go responses induced faster parietal P3 than Nogo responses. For parietal P3 amplitudes, the main effect of Expression was significant (*F*_(1,27)_ = 17.35, *p* < 0.001, *η*^2^ = 0.39, Power = 0.98), and fearful faces induced greater parietal P3 than happy faces. The main effect of Go/Nogo was significant, *F*_(1,27)_ = 9.01, *p* < 0.01, *η*^2^ = 0.25, Power = 0.83, and Nogo responses induced greater parietal P3 than Go responses.

For parietal P3 difference latencies, the main effect of Response condition was significant (*F*_(2,54)_ = 34.89, *p* < 0.001, *η*^2^ = 0.56, Power = 1.00), and the multiple responses and inhibition responses showed larger difference P3 latencies than conflict control response (*p*s < 0.001). For parietal P3 difference amplitudes, the main effect of Response condition was significant (*F*_(2,54)_ = 4.03, *p* < 0.03, *η*^2^ = 0.13, Power = 0.67), and the *post hoc* analyses showed that multiple responses had more positive difference P3 amplitudes than conflict control (*p* < 0.05).

## Discussion

The current study investigated the interaction between response inhibition and conflict control on facial expressions. It was found that the identification of happy faces was more accurate in the congruent trials than incongruent trials. During neural processing of monitoring process, Nogo responses induced faster and stronger detection processing compared to Go responses; for Nogo responses, incongruent trials elicited greater detection process than congruent trials. During the neural processes of conflict resolution/inhibition control, the valence of the expressions could modulate the frontal P3 responses in response inhibition but not in conflict control. Taken together with the difference waveform analyses, the current findings showed that response inhibition and conflict control on emotional information relied on distinct processes.

Conflict control on emotional faces mainly focuses on the detection and resolution on affective conflicts (Albert et al., 2010; Bayle and Taylor, 2010; Etkin et al., 2011). In line with prior studies, it was currently observed that the response speed was faster to identify happy faces than fearful faces (Leppänen et al., 2003; Leppänen and Hietanen, 2004; Schulz et al., 2007). In addition, participants performed with higher accuracies when identifying happy faces in congruent trials (HHHHH) than in incongruent trials (FFHFF), which was consistent with previous findings of conflict control on emotional information (Eastwood et al., 2001; Fenske and Eastwood, 2003; Rowe et al., 2007; Ochsner et al., 2009; Liu et al., 2013). Fenske and Eastwood’s (2003) behavioral findings manifested that the flanker-compatibility effect was smaller for negative target faces compared with positive target faces, which illustrated that the constriction of attention could be influenced by the valence of emotional faces. Previous studies have found that cognitive control could be influenced by emotional valence of facial expressions (Fenske and Eastwood, 2003; Liu et al., 2013) and also levels of motivation intensity (Harmon-Jones et al., 2013). Harmon-Jones et al. (2013) demonstrated that the affects of low motivational intensity could broaden the attentional scope, however, the affects of high motivational intensity narrowed the scope. The regulation of emotion when viewing sport video could induce the changes of skin conductance responses (SCRs), and the SRCs were increased in the emotion regulation condition compared with the control condition (Morawetz et al., 2016). In the current study, the emotional expressions were not as arousing as the sport film clips which might only require participants’ recognition of expressions and might only further induce their emotional state changes during the judgment of the expressions. In addition, response inhibition on emotional faces can be attributed as inhibition processes to certain emotion or affective situation, which activates frontal cortex to engage in deliberate top-down attentional control upon emotional context (Nieuwenhuis et al., 2003; Shafritz et al., 2006; Dennis and Chen, 2007; Chiu et al., 2008; Kiss et al., 2008; Zhang and Lu, 2012). Besides emotional faces, Yu et al. (2012) adopted erotic images or painful video clips before a classic stop signal task, and intended to find out whether participants’ cognitive inhibitory control was affected by the emotional processes on the emotional information. The authors observed that emotional processing on the emotional stimuli impaired male participants’ inhibitory control with slower stop signal RT after viewing emotional stimuli compared to the neutral stimuli. Interestingly, when participants were told that their expressions were recorded by a webcam during the experiment, the impairing effect of inhibitory control was eliminated; these significant findings suggested that the interaction between inhibition control and emotional processing might be affected individual’s state of self-consciousness and cognitive load (Yu et al., 2015).

Priori electrophysiological studies have shown that both conflict control and response inhibition processes could elicit frontal N2 responses, and Nogo-N2 in response inhibition related to the detection of required inhibitory response (Falkenstein et al., 1999) and N2 in conflict control was associated with conflict monitoring processing (van Veen and Carter, 2002a; Carter and van Veen, 2007; Folstein and Van Petten, 2008). Similar to Nogo-N2 responses to non-emotional information, the current Nogo-N2 responses to affective faces were also found to be slower than Go-N2 responses, which illustrated that it took more neural processes to detect Nogo signals comparative to Go signals (Albert et al., 2010). Nogo-N2 responses did not vary with the affective valences of faces (Chiu et al., 2008; Albert et al., 2010, 2012; Zhang and Lu, 2012), which further supported that the interaction between response inhibition and emotional process did not occur in the detection stage (N2 responses; Albert et al., 2012). Neuroimaging and brain injury studies showed that the frontal areas (such as the right inferior frontal cortex [rIFC], and ACC) were the fundamental brain regions for general response inhibition (Aron et al., 2004; Hampshire et al., 2010) and also affective response inhibition on emotional faces (Ochsner et al., 2004; Shafritz et al., 2006; Albert et al., 2010). The neural development study using Time-Frequency analysis also found that frontal beta power highly related to the development of inhibition control in 5–6 years old children (Lo et al., 2013). Moreover, Morawetz et al. (2016) investigated the functional interrelationships among several brain regions during emotion regulation by presenting short videos to participants and checking the manipulation with SCRs, and it was found that dorsolateral prefrontal cortex (DLPFC) was the key node of the prefrontal emotion regulation network and strongly connected with the inferior frontal gyrus (IFG).

Neuroimaging studies have shown that bilateral dorsal ACC, posterior medial frontal cortex, and DLPFC were strongly activated to execute affective conflict control (Ochsner et al., 2009), and DLPFC was also activated during threat-related learning (Wheelock et al., 2014). It was also currently shown that the conflict detection of happy faces with fearful distractors induced more negative N2 responses relative to that of fearful faces with happy distractors, and in the same vein Fenske and Eastwood’s (2003) behavioral findings manifested that the flanker-compatibility effect was smaller for negative target faces compared with positive target faces, which illustrated that the constriction of attention could be influenced by the valence of emotional faces (Fenske and Eastwood, 2003). Furthermore, it was currently observed that the monitoring of target happy face induced greater N2 activation in incongruent trials than congruent trials, which replicated the previous findings (Liu et al., 2013). It was further demonstrated that individual’s visual view field was widened by positive information; therefore, it required incremental interference control on happy targets with fearful distractors (Eastwood et al., 2001; Rowe et al., 2007). Unexpectedly, the reverse effect on N2 amplitudes of fearful faces between congruent and incongruent trials was observed compared to that of happy faces. Some relevant ERP and behavioral studies did not observe the significant differences between congruent trials and incongruent trials in response to fearful faces as that to happy faces (Liu et al., 2013; Yang et al., 2016). Yang et al. (2016) regarded the disappearance of emotional conflict effect for negative faces were due to the timely resolution on the negative conflicts. The current results might further reveal that the conflict monitoring was enhanced when the stimuli only contained fearful faces, and the negative fearful information might narrow one’s view field (Fenske and Eastwood, 2003). Taken together, these findings suggested that when the processes required both conflict control and response inhibition, and the congruency effect would be weaken. Compared to conflict control processes, response inhibition processes were likely modulated by emotion.

With regard to P3 responses, P3 activation in response inhibition relates to executive/inhibitory control (Bokura et al., 2001), and P3 in conflict control reflects conflict resolution (Hillman et al., 2009a,b; Clayson and Larson, 2011). Source location analyses showed that prefrontal cortex and parietal areas might be the neural generators of P3, which were responsible for inhibitory control, conflict resolution and outcome valuation processes (Bokura et al., 2001). It was currently found that Go responses to happy faces induced faster frontal P3 than that to fearful faces and frontal P3 Nogo response to fearful faces was faster than that to happy faces. These findings might reveal that it was easier to execute response on happy information than fearful information; however, on the contrary, it was easier to inhibit on fearful information than happy information. Nogo responses induced greater frontal P3 than Go responses, which illustrated that frontal cortex was related with inhibition control processes. In addition, inhibition control on fearful faces elicited greater P3 activation relative to executive control, and Nogo-P3 responses were greater for fearful faces compared to happy faces, which was similar with the findings that response inhibition on negative stimuli required more action inhibitory process (Yu et al., 2014). Moreover, Senderecka (2016) also investigated the interaction between inhibition control and emotional processing on arousal stimuli via a emotional stop signal task, and it was observed that emotional processing on threatening stimuli improved participants’ inhibition control with increasing inhibitory rate and decreasing stop signal RT. Taken together with Yu et al.’s (2012) study, it can be deduced that whether emotional processing impairs or improves the subsequent inhibition control might also be influenced by the valence of the emotional stimuli.

The neural mechanisms of parietal P3 responses were similar with the late positive potential (LPP) during emotion regulation processes which was sensitive to emotional attentional deployment, such as, distraction (Thiruchselvam et al., 2011), attention modulation (Hajcak et al., 2009), and directions to regulate subjective affective responses (Krompinger et al., 2008; Moser et al., 2006, 2009). Moreover, fearful faces with happy distractors required greater conflict resolution processing (longer and greater P3 responses) compared to fearful faces in congruent trials, which was consistent with previous findings (Liu et al., 2013) and might reveal that affective incongruent trials required more attentional control process on the conflicts compared to affective congruent trials (Liotti et al., 2000; Frühholz et al., 2011).

Importantly, the difference analyses revealed that the multiple processes and response inhibition induced stronger N2 and P3 responses than conflict control processing, and this finding manifested that the multiple emotion regulation processes required more neural efforts than the comparatively simple conflict control processing. Furthermore, these current findings might indicate that response inhibition and conflict control on emotional information were two distinct processes, which was consistent the findings in the non-emotional contexts (Bunge et al., 2002; Brydges et al., 2012, 2013). The separation between interference control and response inhibition has been investigated via the non-emotional Go/Nogo flanker task (Brydges et al., 2012, 2013), and participants were instructed to respond according to the color and direction of the stimuli (fishes). It is reported that the incongruent flanker condition induced a larger and later N2 responses compared with the Nogo condition, indicating the separation of interference control and response inhibition. In the same vein, Bunge et al. (2002) adopted the arrow flanker and Go/Nogo task in children, and it was observed that children experienced difficulty in suppressing inappropriate responses and were more susceptible to interference. Children’s effective interference control was related with prefrontal activation and their effective response inhibition was associated with activation of posterior brain regions. Therefore, from the neurodevelopment perspective, the conflict control and response inhibition processes required varied brain activities since the childhood.

There were contrary opinions on whether dissociable brain mechanisms were involved in emotional and non-emotional cognitive control. Some studies demonstrated the domain-general control network for both emotional and non-emotional control, which observed the similar activation of ACC and DLPFC during conflict control on both emotional and non-emotional stimuli in the revised AX Continuous Performance Task (AX-CPT; Chiew and Braver, 2011). However, some studies held the opposite opinion that brain activated distinct brain networks for emotional and non-emotional control on conflicts, and the dissociation also occurred on the brain function level (Egner et al., 2008; Soutschek and Schubert, 2013; Mian and Eskandar, 2016; Xue et al., 2016). By using emotional and non-emotional stroop tasks, Egner et al. (2008) illustrated that cognitive control on non-emotional conflicts activated dACC and DLPFC for conflict monitoring and attentional control, respectively; while, for emotional conflict control processing, dACC and amygdala were enhanced for conflict monitoring, and rostral ACC instead of DLPFC was increasingly activated for attention control on conflicts. Xue et al. (2016) also reported the differences between emotional and non-emotional cognitive control processes with larger conflict effect (RT in the incongruent trials minus RT in the congruent trials) in non-emotional than emotional stroop tasks. Besides, Ochsner et al. (2009) using emotional and non-emotional flanker tasks to compare the neural activation differences, and it was observed that DLPFC, dorsal ACC and posterior medial frontal cortex were activated during conflict control on both emotional and non-emotional conflicts; however, rostral medial PFC and left ventrolateral PFC were separately activated during affective and cognitive control processes. Therefore, Ochsner et al. (2009) further indicated that conflict control on emotional and non-emotional conflicts depended on shared and distinct brain systems. With regard to inhibition control on affective information, Depue et al. (2006) revealed that inhibitory effects were larger for emotional information than non-emotional information when investigating suppression in memory. As for the interaction processes between responses inhibition and conflict control, it required further exploration to identify the differences in emotional and non-emotional contexts in the future study. In addition, it has been reported that individuals showed perceptual expertise for own-related faces (Wiese, 2013) and they could remember own-gender faces more accurately than other-gender faces (Wolff et al., 2014). However, we did not adopt large amount of trials to investigate the own-gender bias in the present study, and in our future work, we would increase the numbers of trials and participants to further explore own-related bias when investigating the interaction between response inhibition and conflict control processes.

In summary, the current study investigated the interactions between responses inhibition and conflict control on affective information with the emotional Go/Nogo flanker task. The electrophysiological findings showed that the interaction between these two emotion regulation processes mainly occurred during monitoring/detection stage, and the multiple processes and response inhibition on emotional information required more neural efforts than conflict control processing. The current study also suggested that response inhibition and conflict control required distinct neural network activities in the emotional context.

## Author Contributions

TL and JS designed the experiment. TL and TX collected and analyzed the data. TL, TX and JS wrote the manuscript.

## Conflict of Interest Statement

The authors declare that the research was conducted in the absence of any commercial or financial relationships that could be construed as a potential conflict of interest.
